# Cultural Attachment: From Behavior to Computational Neuroscience

**DOI:** 10.3389/fnhum.2019.00209

**Published:** 2019-06-20

**Authors:** Wei-Jie Yap, Bobby Cheon, Ying-yi Hong, George I. Christopoulos

**Affiliations:** ^1^Nanyang Business School, Nanyang Technological University, Singapore, Singapore; ^2^Decision, Environmental and Organizational Neuroscience Lab, Culture Science Institute, Nanyang Technological University, Singapore, Singapore; ^3^School of Social Sciences (Psychology), Nanyang Technological University, Singapore, Singapore; ^4^Singapore Institute for Clinical Sciences, Agency for Science, Technology and Research (A^∗^STAR), Singapore, Singapore; ^5^Department of Marketing, The Chinese University of Hong Kong, Hong Kong, Hong Kong

**Keywords:** cultural attachment, attachment, neuroscience (psychology), symbols, culture

## Abstract

Cultural attachment (CA) refers to processes that allow culture and its symbols to provide psychological security when facing threat. Epistemologically, whereas we currently have an adequate predictivist model of CA, it is necessary to prepare for a mechanistic approach that will not only predict, but also explain CA phenomena. Toward that direction, we here first examine the concepts and mechanisms that are the building blocks of both the prototypical maternal attachment as well as CA. Based on existing robust neuroscience models we associate these concepts and mechanisms with bona fide neurobiological functions to advance an integrative neurobiological model of CA. We further discuss the unresolved relationship of CA to other similar socio-cognitive concepts such as familiarity. Overall aim of the paper is to highlight the importance of integrating CA theory to computational approaches to culture and evolution (such as predictive processing computations explaining niche construction), as this will allow a dynamic interpretation of cultural processes.

“One might almost say that a culture is to a society,

as the memory is to a person”

Kluckhohn, 1954, pp. -694–695

Javaria, a South-East Asian student in the US, was willing to travel all the way from Massachusetts to New York to find the desi food that reminds her of home (true story: Khan, 2012). A soldier posted overseas carries a flag under his uniform. A student preparing for her exams listens to a playlist with songs from her country. A suicide bomber reads holy scripts as a preparation for his mission (Glucklich, 2009). People demonstrate against their childhood playground being demolished to be replaced by an undoubtedly needed and high return-on-investment mall. People are unwilling to leave their old houses, even if offered a modern apartment in exchange. Listening to the national anthem increases heart rate. Even on the verge of bankruptcy, Greeks will nevertheless strike and shut down Acropolis declaring it is “not for sale” – as a response to rumors that Acropolis is part of the bailout deal (Associated Press, 2018).

These real life examples, which are far from unique and experienced by many all over the world, characterize the relationship between individuals and aspects of their culture – such as food, flags, songs, monuments, and spaces. Despite their relevance to culture, these examples cannot be accounted for by some major theories in psychology, such as Hofstede’s cultural dimensions (Hofstede, 2011), or Triandis’ individualism–collectivism (Triandis, 2010 for review). It is because these major theories view cultures as static entities for assessment, rather than cultures as functional entities that meet individuals’ psychological needs. Moreover, to the extent that these research focus on aggregated tendencies of cultural or national groups, it is uncommon for this approach to integrate biological mechanism into their theories. The recently emerged field of cultural neuroscience has potential to fill this gap (e.g., Chiao and Ambady, 2007). However, most of the research in this nascent field still focuses on mapping out neural correlates for cross-cultural differences. By contrast, theories originated from the disciplines of anthropology and cultural studies have focused more on how culture evolves through people’s daily practices and experiences (see reviews by Shweder, 1999; LeVine, 2010). Yet, most of these theories have not addressed how biology underlies people’s cultural experiences. The goal of this paper is to fill this knowledge gap. In short, we take a functional perspective by analyzing cultural experiences through the lens of “cultural attachment” (CA), addressing an individual’s bond with culture, and suggesting how that bond is mediated by biological mechanisms.

To elaborate, the aforementioned examples suggest that cultural objects carry an emotional value as well as meanings that are learned. Indeed, scholars have previously highlighted the importance of affectional bonds between the individual and other members of the cultural group (Chao et al., 2015) and also shown that culture is held by mental memory networks which become accessible when primed (Hong et al., 2000; Hong and Khei, 2014). We here examine this set of psychological phenomena that underlie the dynamic relationship between culture, its symbols and individuals, at a behavioral, neural and computational level. Specifically, we contend that culture – defined as a network of beliefs, values, practices, and meaning system that are shared and transmitted from generation to generation among members of a group – can serve as a secure base for attachment. That is to say, individuals can form a secure bond with their culture, which in return can buffer threat and confer a feeling of safe haven for individuals. This phenomenon has been called “cultural attachment” (see review by Hong, 2017). In this paper, we delve into an under-explored aspect of CA – What are the possible physiological and neurological processes underlying CA? To address this question, we will focus on how CA moderates the processes of threat appraisals, emotion regulation and coping, and how the human neurobiology supports these processes.

As with any theory in its early stages, it is essential to prepare the ground for a move from a predictivist model of CA to mechanistic approaches. A predictivist approach will rely on statistical regularities to ascertain the existence of a phenomenon – in this case the function of culture as an attachment object – and will develop models that should be able to make (accurate) predictions. However this would only mean that the model is merely phenomenal (characterizing without explaining), whereas a mechanistic approach would necessitate explanatory models that not only predict the phenomenon but as well unveil the underlying mechanisms. This is vital, as predictively adequate models might actually fail at an explanatory level. We explain: a mechanistic approach would involve describing the phenomenon by elaborating on its components, the associated functions and the way they are structured together – for instance in terms of space, time or hierarchy (see Bechtel and Richardson, 1993; Machamer et al., 2000; Craver, 2007). This means, amongst others, to involve neurobiological components and have a model that “address the question of how nervous systems operate on the basis of known anatomy, physiology, and circuitry” (Dayan and Abbott, 2001, p. xiii). Undoubtedly, this is an ambitious target for CA theories at this current stage; yet, it is important to organize the predominantly predictivist character of the current understanding and prepare for more mechanistic accounts. To that end, we can take advantage of the existing, relatively rich, corpus of cognitive neuroscience to map CA components to neurobiological mechanisms. This is one of the main targets of this paper.

The paper is structured into two major sections. In the first section, we review current literature in AT and CA before proposing a mechanistic neurobiological model of CA. In particular, we focus on the components in the model (internal working model or IWM, threat processing, cultural stimuli acting as secure bases) and dedicate separate sections reviewing findings that correspond to these components. Following that, we integrate each component into the final model by enlisting psycho-cognitive processes needed to generate these CA functions and by identifying the neurological processes that – based on previous studies associating cognitive processes to neuronal signatures – reflect these situations. In the second section, we discuss two open questions that future studies need to explore: (1) the relationship of CA not only with prototypical attachment but other similar concepts (such as familiarity and group identity) as well, and (2) the need of and steps toward the adoption of computational approaches to the understanding of CA – i.e., a mechanistic epistemological framework.

## Cultural Attachment: an Extension of Attachment Theory

CA was developed as an extension of Attachment Theory (AT) because the acculturation process bears similarities to an infant being born (Hong et al., 2006, 2013). When an individual is introduced to a new culture, he/she is introduced to different norms, values, beliefs and structures. The home culture (i.e., predominant culture where the individual spend most of their growing years in) is believed to act as a secure base allowing the individual to explore and better regulate his/her emotions in the new environment (Fu et al., 2015; Hong et al., 2016). In fact, there is initial evidence that maternal, paternal, and CA affect different aspects of intercultural adjustment (Phua et al., 2017).

AT was initially developed by John Bowlby and Mary Ainsworth to describe the relationship and bond between a child and his/her caregivers and how that relationship affects the child’s psychocognitive development (Bowlby, 1969; Ainsworth and Bowlby, 1991). Attachment refers to the unidirectional (and only sometimes reciprocal) emotional bond that an individual has to an attachment target. Traditionally, this target would usually refer to the primary caregiver of the child – the mother. Yet, in later developments of the theory, researchers have extended the scope to other targets such as significant others, countrymen, non-human animals or even non-living objects (Keefer et al., 2014).

**FIGURE 1:**
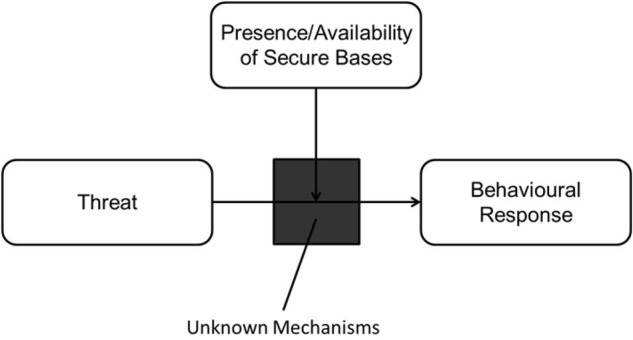
A general model of attachment.

Over the last 40 years, AT has significantly contributed to our understanding of the social and emotional development of humans. The reach of AT is widespread and involves many different fields, including developmental psychology, evolutionary psychology, social psychology, neuroscience, psychophysics, and ethology. Detailed progress behind the theory and its historical timeline is described in several comprehensive reviews (Bretherton, 1985; Mikulincer and Shaver, 2003; Bowlby, 2008; Bretherton and Munholland, 2008; Cassidy and Shaver, 2016). It is currently unknown if all the features and major tenets of AT also apply to CA. For the purposes of this paper, we focus on two of the many tenets established in AT: the IWMs and the function of attachment targets as secure bases. Figure 1 shows a simplified model of attachment containing these two tenets. The IWM (the black box), through repeated experiences, identifies and selects reliable attachment targets which then act as secure bases when these individuals are under threat. The mechanisms behind the IWM and its interaction with the presence and availability of secure bases are currently under-explored. In the following sections, we will review the current state of research and begin development of a cognitive framework for these two tenets of AT and CA.

The pursuit of identifying the presence and nature of a cognitive framework is difficult without first establishing biological differences in threat responses due to CA. The effectiveness of culture as a secure base has been examined using behavioral experiments (Hong et al., 2013) but more similarly robust results are needed to present a convincing case. As converging, multi-modal evidence heavily reduces the possibility of spurious findings, psychophysiological and neuroscientific methods can be employed to examine if the phenomenon occurs at a more subconscious level, as well as to identify brain areas or networks contributing to IWM. In the sections which follow, we summarize the current understanding of the cognitive and affective elements of attachment as well as detail possible neurological mechanisms.

## Attachment Theory: Internal Working Models

An IWM refers to the cognitive framework comprising of mental representations based on the individual’s experiences (Piaget, 1952; Bowlby, 1969, 1973, 1980). In AT, IWMs relate to representations of the self, others, and their relationship. These representations are mostly implicitly learned and based on childhood experiences with the caregiver. This relationship generates the first rules and expectations about how to interact with others. These rules are then transferred to other social relationships. The way early IWMs are developed and later influence adult life is the core of the prototypical (maternal) AT. Early experiences with the caregiver form a basis for an IWM of social relationships. As children interact with their caregivers, their experiences accumulate and expectations about each role (e.g., the self, the caregiver, and the accessibility and responsiveness of the caregiver) are constructed (Bowlby, 1969). In later social interactions, memories and information processing frameworks formed from previous interactions provide the child with “guidelines” (Bowlby, 1969; Bretherton and Munholland, 2008), even as the child progresses into adulthood (Hazan and Shaver, 1987). IWMs developed during the age period of 2–6 years are reportedly sophisticated; schemas about others are fairly accurate; overall, children are able to develop an understanding of precise and complicated rules of social interactions (Nelson and Gruendel, 1981; Greenberg and Marvin, 1982; Main et al., 1985; Stern, 1985; Slough and Greenberg, 1990; Cassidy et al., 1992; Bretherton, 1993; Dunn, 1993; Miljkovitch et al., 2004; Waters and Waters, 2006).

IWMs are dynamic across time and need to be updated to ensure their stability and continuity (Bowlby, 1969, 1982; Bretherton and Munholland, 2008). In fact, some researchers have associated IWMs with the development of brain areas related to memory and socio-cognitive capabilities (Thompson, 2000, 2006). Examples include the emergence of Theory of Mind, where the child is able to attribute mental states to others; or event representations of episodic memories, where the child remembers and recalls details of separate events. As the brain develops, representations are also updated and the number of different ways of understanding the same experience increases. For example, secondary representations of experiences, mediated by language or affect, change as the capabilities of the child improves. In fact, attachment attitudes and styles of preschool children are better predictors of adult attachment than their infant attachment styles (Thompson, 2006, 2015). Researchers have also suggested that the attachment structure of adults in their middle or later stages of their life is different from younger adults. Specifically, a principal component analysis performed on the Relationship Style Questionnaire revealed seven factors in samples of older participants in comparison to the three that were originally proposed (Magai, 2008). This suggests that IWMs become more complex and nuanced as individuals age.

However, once IWMs stabilize, they are less malleable. Bowlby (1973, 1980, 1988) believed that once defensive mechanisms are established in a child’s IWM, changes are difficult to instill. This is supported by selective processing by insecurely attached adults, where they have a tendency to suppress or exclude attachment-related information regardless of the valence of the information (Mikulincer and Shaver, 2007a, 2011; Dykas and Cassidy, 2011). Critically, only attachment related information seems to be consistently overlooked whereas general information processing was not affected (Mikulincer and Shaver, 2007b). Other researchers revealed that securely attached children generally paid more attention to positive events (Belsky et al., 1996). This suggests the possibility of inertia in the initial state of the IWM. Securely attached individuals are in a positive cycle to seek and absorb attachment related information whilst insecurely attached individuals are in a negative cycle to reject and avoid attachment related information. On top of that, attachment is naturally a dyadic bond. In long lasting relationships, the IWMs of both parties have set expectations of the other. Changes in the behavior of one party may be met with resistance from the other party resulting in the maintenance of the status quo.

Finally, stable and continuous attachment relationships are likely to result in strong secure attachment bonds (Egeland et al., 1990; Sroufe et al., 1990; Belsky and Fearon, 2002). Strong secure attachment bonds, once formed, are not easily displaced. For example, an individual is unlikely to completely lose faith in the attachment figure just because the emotional sensitivity of the attachment figure lowers for a few interactions. In line with Piaget (1976) idea of assimilation, these individuals are not alarmed by sporadic lapses in consistent behavior from the attachment figure. On top of that, familiar relationships demand less attention and are more likely to be automated. This is in line with the idea that individuals prefer to maintain familiar relationships to reduce the cognitive effort needed for social interaction otherwise (Coan and Sbarra, 2015). However, this lack of attention leads to the slow updating of IWMs.

It is apparent that IWMs are carriers of culture. Culture needs to be learned, even if we assume that some aspects of culture are genetically carried across generations. At a very fundamental level, humans, and potentially some non-human primates, need to learn the shared meanings, expectations and, generally, “ways of life” of their group. The theory of CA suggests that, similar to attachment bonds to caregivers and other adults, CA provides psychological security to aid in the defense of imminent threats (Hong et al., 2006, 2013; Hong, 2017). CA follows a two-pronged process in which individuals are attached to the members of the cultural group and to the abstract symbolic representations of the culture. Based on evidence that children and adults are attached to multiple targets – including other family members (Weisner et al., 1977; Puddy and Jackson, 2003), romantic partners (Hazan and Shaver, 1987; Mikulincer et al., 2001a), organizational members (Davidovitz et al., 2007), fictional characters, pets (Sable, 2013), deities (Kirkpatrick, 2005; Granqvist et al., 2007), places (Scannell and Gifford, 2010, 2017; Lewicka, 2011), and even objects (Keefer, 2016) – the cultural group and its associated symbols can be viewed as an extended group of caregivers and attachment targets. It needs to be emphasized that, at the individual level, CA patterns do not necessarily reflect, or are the outcomes of, attachment bonds toward caregivers.

Yet, culture is a far more complex network of shared meanings (Hong, 2009) than family; this means that culture is difficult to represent in the same way as typical attachment figures (such as the mother) are mentally represented. In other words, some aspects of CA have to be formed based on the intangible nature of culture (Becker, 2013; De Berker et al., 2016). The cognitive framework of CA, predominantly based on symbolic representations such as flags, objects, places, statues etc., could be activated when such symbols are displayed (Hong et al., 2000). For example, the term “motherland” itself could act as a mnemonic for the nation (Diener, 2002). In order to reliably activate this cognitive network, a dynamic constructivist approach to culture is needed. Under the dynamic constructivist approach, an associative memory network containing culturally relevant information (such as norms, beliefs, and values) can be activated to influence cognition and consequently, behavior (Briley et al., 2000; Hong et al., 2000; Briley and Wyer, 2001; Hong, 2009; Wyer, 2014). As such, although not formally stated, it can be argued that a dynamic constructivist view of culture consists of IWMs of culture.

Both theoretically and methodologically, the processes of cultural priming can to some extent exemplify the dynamic constructivist approach. Priming an individual involves using stimuli or tasks to activate mental representations to serve as frames of references for future tasks (Higgins, 1996; Bargh and Chartrand, 2000). Various types of priming, using a range of mediums (such as words, images, or other sensory information), are used to achieve different goals. In general, perception of certain stimuli will activate networks related to that stimuli and lead to behavior congruent to that of the stimuli (Dijksterhuis and Bargh, 2001). For example, sales of French and German wines in a supermarket fluctuated depending whether French or German music was being played (North et al., 1999).

For culture research, more emphasis is placed on the individual’s acquisition and application of cultural knowledge system, or dual cultural knowledge systems in the case of bicultural individuals. Cultural priming involves increasing the accessibility of a particular cultural knowledge system acquired by individuals by using culturally relevant stimuli such as images unique to that culture, words commonly associated with that culture or a language primarily used in that culture (Trafimow et al., 1991; Hong et al., 2000, 2013; Briley and Wyer, 2001; Ross et al., 2002; Verkuyten and Pouliasi, 2002; Briley et al., 2005; Chen et al., 2005; Fu et al., 2007a,b; Briley, 2008). This is evident from research in bicultural individuals (who have been extensively exposed to two cultures) who displayed ability to – consciously or not – switch cultural frames when making decisions (Hong et al., 2000, 2003). A natural extension of the dynamic constructivist approach is the development of polyculturalism which posits that cultural traditions are shared amongst different cultures, and individuals are partially influenced by different cultures (Morris et al., 2015). Despite the many cultural constructs or networks of information, priming makes a specific cultural construct accessible to the individual and influences decision making in contexts where the construct is applicable (Hong et al., 2003). For example, it was shown that Chinese-American bicultural individuals were more cooperative toward ingroup partners in a Prisoner’s Dilemma Game when primed with Chinese cultural icons than when primed with American cultural icons. This difference was mediated by a higher motivation to maximize joint outcomes with partners and higher expectation that the ingroup partners would cooperate in the Chinese priming than in the American priming condition. However, this difference between the two cultural priming conditions disappeared when the partners in the game were strangers because strangers were not expected to cooperate more in Chinese culture and thus the activated motivation to cooperate did not apply anymore (Wong and Hong, 2005).

In the development of CA, particular focus was placed on bicultural individuals – individuals who have been exposed to two cultures extensively. Using cultural priming experiments involving bicultural individuals, researchers (Trafimow et al., 1991, Hong et al., 1997, 2000; 2003; Ross et al., 2002; Verkuyten and Pouliasi, 2002; Wong and Hong, 2005; Elaine Perunovic et al., 2007; Fu et al., 2007a,b; Sui et al., 2007; Ng and Lai, 2009; Chiao et al., 2010; Tam et al., 2012) were able to examine the fluidity of cultural frames and the existence of separate cognitive frameworks for each culture. Different cultural symbols seem to be capable of activating their own respective frameworks, supporting the notion that there is a mental representation network associated with each culture. These mental representation networks possibly form part of an IWM. Similar to arguments made in polyculturalism, individuals can be attached to more than one culture and secure attachment toward either culture aided in predicting that individual’s levels of resilience toward threats (Hong et al., 2013).

## The Neurobiology of Internal Working Models

Not surprisingly, given the complexity of attachment, dedicated neural circuits of attachment have not been isolated and in fact, are posited to be inexistent (Coan, 2008, 2016). Instead, researchers suggest that attachment is a higher-order culmination of different existing networks in the brain (Coan, 2008, 2016). This is in line with Bowlby (1969) description of attachment being a “biobehavioral state in which several physiological and behavioral systems are organized in order to provide an individual with a certain sense of security and intimacy with significant others” (c.f. Gander and Buchheim, 2015, p. 1). Similarly, due to the multi-determined nature of psychophysiological measures, dedicated circuits for bodily responses reflecting attachment bonds have also not been identified (Fox and Hane, 2008; Buchheim et al., 2017). However, several reviews bridging research in attachment to the brain and the body (e.g., Coan, 2008, 2016; Vrticka and Vuilleumier, 2012; Gander and Buchheim, 2015) have proposed possible models of how attachment functions. In particular, the two models (Vrticka and Vuilleumier, 2012; Coan, 2016) detailed how attachment is represented in the brain both highlighted the cognitive and affective elements of attachment and structured their models around the corresponding areas in the brain.

Attachment engages two different types of components in the brain: affective and cognitive (Vrticka and Vuilleumier, 2012; Canterberry and Gillath, 2013). IWMs can be thought of as the cognitive component of attachment capturing information about interactions between the individual and the attachment target. Yet, as it will become apparent below, it is practically impossible to separate cognitive and emotional elements. These frameworks were suggested to be conditioned associations between the availability or responsiveness of attachment figures (or proximity) and the internal needs or external threats mediated through different regions in the brain such as amygdala, nucleus accumbens (nACC), hippocampus, and prefrontal cortex (Hofer, 2006).

**FIGURE 2:**
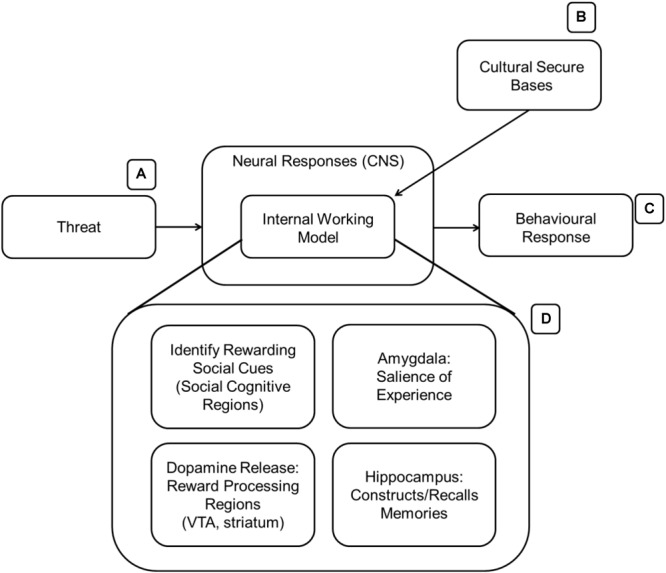
The presence of culturally relevant stimuli **(B)** activates the IWM **(D)**. The expanded version of the IWM contains several brain regions that could be involved in acquiring and learning about valuable cultural stimuli. AT suggests that the IWM is activated in the presence of threat **(A)** to aid in mitigation before a behavioral response **(C)** is made.

Coan (2016) proposed an example of how different networks in the brain would work together to identify, maintain and benefit from attachment bonds (see Figure 2). Many parallels can be drawn between the suggested model and the functioning of an IWM. Reward processing regions of the brain [such as ventral tegmental area (VTA), nACC, and the striatum overall] are activated (possibly be predominantly involving dopamine release) when in contact with a secure attachment target; moreover, the amygdala tags the experience as emotionally salient before long-term memory consolidation in the hippocampus. As multiple memories form, a consolidated semantic understanding of the attachment target is formed and used whenever primed or reminded of him/her/it. Upon recall, the same reward processing networks release dopamine again aiding in emotion regulatory regions (such as various parts of PFC, ACC) in exercising top down regulation of affective regions of the brain experiencing threat or other negative emotions. Social cognition regions (see below) of the brain contribute to this process by identifying “rewarding” attachment targets and by promoting behaviors that outsource the cognitive load of emotion regulation to them. At the same time, the brain reinforces rewarding choices and avoids negative ones.

Memory processes provide a framework for information obtained in past interactions to be used in future ones. Memories, typically differentiated by their duration, serve various functions. Episodic memory contains personal experiences while semantic memory contains factual knowledge (Tulving, 1972). Experiences between different attachment targets are encoded as memories and integrated to form a coherent IWM (Bretherton, 1990; Thompson et al., 2003; Mikulincer and Shaver, 2004).

The hippocampus plays an important role in the formation, storage, consolidation and retrieval of internal and external states (Brasted et al., 2003; Kennedy and Shapiro, 2004). Additionally, the amygdala also plays an important role in the consolidation of positive and negative long term memory by tagging sensory experiences as significant or salient before the consolidation of long term memory. Amygdala activity has also been detected when emotionally salient information is being recalled weeks after exposure (Hamann et al., 1999). These two regions in the brain have been implicated in various attachment studies. The interaction between amygdala and locus coeruleus facilitates familiarity and reinforces filial bonds. Norepinephrine from the locus coeruleus, which neonates release in large quantities (Nakamura and Sakaguchi, 1990), is necessary for learning in human and animals (Sullivan, 2003) as well as memory consolidation specifically (Cahill et al., 1994), especially in the amygdala (Liang et al., 1995; Kodirov, 2012). Individuals with high attachment avoidance, similar to patients with PTSD, have low cell density in their hippocampus (Quirin et al., 2009) suggesting that attachment avoidance is related to selective formation of memories. Culture, when viewed as a culmination of knowledge across a group or a mental framework of norms, shared meanings and associated behaviors, necessitates a memory capacity of some sort to hold this knowledge or framework. One can argue that without memory processes, culture as we know it will cease to exist.

IWMs engage brain areas involved in reward processing, mostly part of the dopaminergic system such as VTA and nACC. Brain areas involved in reward processing (i.e., part of the dopaminergic system – e.g., VTA and nACC) are recruited to identify and evaluate potentially beneficial and desirable attachment targets. Previous research using functional Magnetic Resonance Imaging (fMRI) investigating various forms of love identified activations in reward-related regions – VTA and caudate (Bartels and Zeki, 2000, 2004; Aron et al., 2005; Ortigue et al., 2007). Viewing images of loved ones activates similar areas (such as the caudate head and nACC) as well as other areas such as, lateral orbitofrontal cortex (OFC), amygdala, and dorsolateral prefrontal cortex (Younger et al., 2010). Thus, it could be expected that cultural cues that prime a related cultural network could also activate the same reward processing regions.

## Attachment Theory: Secure Bases

The idea that attachment targets could act as secure bases was first introduced by Ainsworth (1963) after observing interactions of children and their caregivers in the “Strange Situation” – a laboratory procedure designed to systematically measure attachment and exploratory behaviors in infants (Ainsworth and Wittig, 1969). “Secure base schemas” refer to a set of expectations about the accessibility and responsiveness of the attachment target (Sroufe and Waters, 1977a; Waters et al., 1998; Mikulincer et al., 2001b). These schemas set up if-then conditions that provide individuals with scripts (e.g., “If I am threatened, I can approach this person and he/she will be there for me”) when they are under threat. These scripts are posited to create a sense of felt security (Sroufe and Waters, 1977a; Waters and Cummings, 2000; Waters and Waters, 2006). In childhood attachment, secure bases act as anchors for children to explore their environments and a safe haven to retreat to when threatened (Ainsworth et al., 1978; Waters et al., 1998; Feeney, 2004). A secure attachment is a necessary condition for the attachment target to act as a secure base. In the case of a caregiver-child relationship, the child must sufficiently trust that the caregiver will be physically accessible and emotionally available before the child would view the caregiver as a secure base. Other researchers have also described secure bases as a manner of social regulation on emotional processing (Beckes and Coan, 2015). Similar results were also found in adult studies (Mikulincer et al., 2001a, 2002b). Additionally, these “if-then” scripts also allow for symbolic representations of the secure base to grant a similar safe haven and reduce the anxiety of the individual (Mikulincer et al., 2001b, 2002a). Social bonding research also contributes by suggesting that humans tend toward affiliation (Depue and Morrone-Strupinsky, 2005) and recognize signals of social closeness readily (Laird and Strout, 2007; Rolls, 2007).

Several experiments using an affective priming paradigm, where participants are first threatened and then exposed to their secure base, have shown that symbolic representations (such as a painting of a mother holding a child or a well-known cultural landmark) are also effective at mitigating responses to threatening stimuli (Banse, 1999, 2001; Mikulincer and Shaver, 2001; Mikulincer et al., 2001b, 2002b; Beckes et al., 2010; Eisenberg et al., 2011; Hong et al., 2013). This is an important finding because it introduces a way to systematically test the efficacy of attachment in adults and aids the extension of attachment to other domains, as cognitive representations introduce a meaningful way to discuss attachment to in-tangible aspects. This could include the cases where attachment targets are sometimes abstract or imaginary.

Other experiments also examined the efficacy of having secure attachments and secure bases. Securely attached individuals have a lower tendency to perceive stressful situations as threatening (Mikulincer and Florian, 1995). Additionally, they are more open to seeking help when feeling distressed (Mikulincer et al., 2009). There is a higher willingness for securely attached individuals to trust others, even in previously damaged relationships (Mikulincer, 1998) or with members of an out-group (Mikulincer and Shaver, 2001). Securely attached children with better quality of interactions with their caregivers tended to develop better emotional understanding (Laible and Thompson, 1998; Steele et al., 1999; Ontai and Thompson, 2002; Raikes and Thompson, 2006; Thompson and Meyer, 2007; Beckes and Coan, 2015). In the same vein, daily conversations with children about their experiences improve emotion regulation as well (Wareham and Salmon, 2006). Cassidy et al. (1996) showed that securely attached children ascribed benign motives to story characters whilst insecurely attached children inferred hostile intent. Secure attachment also exhibited links with higher self-esteem (Harter and Pike, 1984; Kerns et al., 1996; Sharpe et al., 1998; Doyle et al., 2000; Verschueren and Marcoen, 2002, 2005; Cassidy et al., 2003; Yunger et al., 2005), higher social competence (Denham et al., 2001, 2002; Schneider et al., 2001; Kerns et al., 2007), and more positive self-concept (Cassidy, 1988; Verschueren et al., 1996; Doyle et al., 2000). Similar trends are also present in research of rhesus monkeys showing that their young explore more in the presence of their mothers as well and return to their mothers when frightened (Harlow and Harlow, 1965; Dienske and Metz, 1977; Simpson, 1979).

Culture is posited to have existed since early humans banded together in groups of varying sizes to become more effective and efficient at ensuring their individual and collective survival. The formation of cultural groups also helped to reduce physical and social environmental uncertainty (Grieve and Hogg, 1999; Christopoulos and Tobler, 2016). Important elements of culture (e.g., knowledge, shared meanings, unique languages, practices, social rules, and expectations) can facilitate and speed up the exchanges of people who need to frequently interact, share resources, and swap tangible and intangible assets. Furthermore, repeated interactions between members of the same group lead to the predictability of behavior and expectations, which in turn aid in future interactions. Some of the other functions of culture include differentiated roles, providing social support but also social control, sharing cognitive orientations and goals, as well as regulating affective expressions (Kluckhohn, 1954). Cultures ascribe meanings and frameworks to understand the world, together with a sense of epistemic security and certainty to its members (Pyszczynski et al., 2004). Individuals form emotional connections with other members in their social group, the social group itself and eventually its abstract representations (Smith et al., 1999). Cultural groups protect against outgroups and other forms of threats. The security provided goes beyond the physical aspects and stretches into the emotional aspects as well (Chao et al., 2015). As such, culture can be interpreted to act as a secure base to individuals.

## The Neurobiology of Secure Bases

**FIGURE 3:**
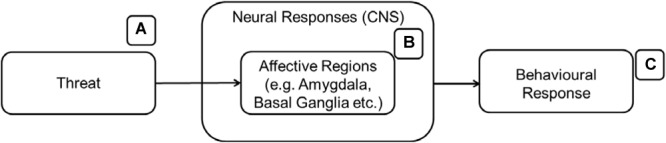
The affective regions of the brain **(B)** are activated when an individual encounters threats **(A)** leading to behavioral responses **(C)**.

The emotion regulatory aspects of attachment have been an important focus for many researchers. For example, Vrticka and Vuilleumier (2012) summarized the attachment system as an emotion regulation device while other researchers viewed it as a major stress regulatory system (Taylor et al., 2000, 2004). Since the presence of threat is a necessary condition to activate cultural stimuli as secure bases, we briefly describe the neurobiology of threat-related responses here. The presence of threat typically activates affective regions in the brain such as the amygdala and the basal ganglia (Ohman, 2005; Figure 3). These affective regions will signal other appropriate brain regions in the brain to prepare the body for a response to the threat. Bodily responses are also commonly measured in the study of threat and are included in some of the research cited in this section. The theory behind the inclusion of bodily responses to the model will be discussed in the following section.

A common finding of studies examining skin conductance responses (SCR) differences between individuals with different attachment styles is that individuals with secure attachments cope with stress better, especially when cognitively reminded or aware of their secure base (see reviews by Diamond, 2001; Gander and Buchheim, 2015). SCR measured during the adult attachment interview (AAI) were higher for participants using deactivating strategies (which implied insecure attachments) compared to securely attached participants (Dozier and Kobak, 1992; Roisman et al., 2004). A similar study measuring attachment avoidance ratings correlated negatively to the SCR of participants under stress, suggesting that even repressed participants were still unconsciously stressed (Diamond et al., 2006). Neural responses of children in the presence of their mother were unaffected by their levels of attachment anxiety. However, when children were left alone children with high attachment anxiety, as compared to children with low attachment anxiety, showed increased activations in ventromedial prefrontal cortex and hypothalamus when presented with threatening words (O’Connor et al., 2012). In comparison to neutral scenes, showing attachment-related scenes from the Adult Attachment Projective (George and West, 2001) activated areas of the brain associated with mental representations and social cognition, such as inferior parietal lobes, middle temporal gyrus, and anterior medial prefrontal cortex (Labek et al., 2016). These studies clearly show the biological effects of successful emotion regulation and threat mitigation in individuals who have access to their attachment targets and are able to leverage them as secure bases.

Various parts of the prefrontal cortex, typically associated with top-down control, support emotion regulation. The medial frontal cortex has been associated with social perception, anticipating the thoughts of others, and predicting outcomes (Amodio and Frith, 2006). The ventromedial and medial orbital areas of the prefrontal cortex have been linked to conditioning and extinction of learning, as well as automatic emotion regulation (Milad et al., 2005; Ellenbogen et al., 2006; Quirk and Beer, 2006; Sierra-Mercado et al., 2006). The dorsolateral prefrontal cortex has been associated with initiating, shifting, inhibiting, and simulating the consequences of behavior (Purves et al., 2008) and linked to several brain areas associated with sensory inputs suggesting that a role in decision making involving multiple sources of information (Miller and Cohen, 2001; Krawczyk, 2002). In contrast to the ventromedial and medial orbital regions, the dorsolateral prefrontal cortex has been linked to more effortful and deliberate emotion control (Ochsner et al., 2002; Sheppes et al., 2009). There is also support for the dorsolateral prefrontal cortex being related to language functions and working memory for representations of up to 10 s (Fuster, 1997; Ochsner et al., 2002; Kanske et al., 2010). The relatively slower development of the prefrontal cortex during infancy has led to suggestions of caregivers acting as a surrogate prefrontal cortex (Gee et al., 2013).

**FIGURE 4:**
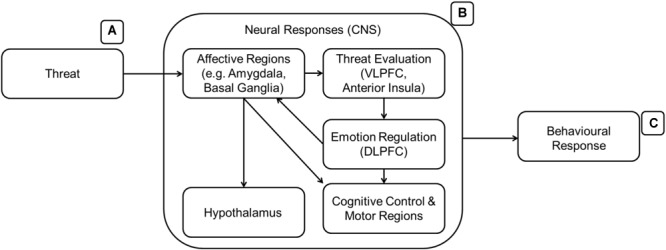
A model of emotion regulation (**B**, Kohn et al., 2014). After the affective regions are activated, the threat **(A)** is then evaluated. Subsequently, the emotion regulation region (DLPFC) is recruited. The activation of the DLPFC signals successful to the affective regions and other regions required for a response **(C)** to the threat if the emotion regulation is not successful.

Kohn et al. (2014; Figure 4) developed a heuristic model of emotion regulation with three main emotion regulation stages. The amygdala and basal ganglia will signal other parts of the brain (ventro-lateral prefrontal cortex – VLPFC, anterior insula, SMA, angular gyrus, STG) upon arousal. The VLPFC will appraise the emotion and decide if there is a need for regulation. If regulation is needed, the emotion regulation process will engage the DLPFC to communicate with the other regions of the brain that will generate the emotional state and behavioral responses. In the case of CA, the DLPFC or other regions should react differently only toward cultural symbols and in the face of threat. Figure 4 details the emotion regulation process which involves the affect processing regions from the previous figure (Figure 3).

**FIGURE 5:**
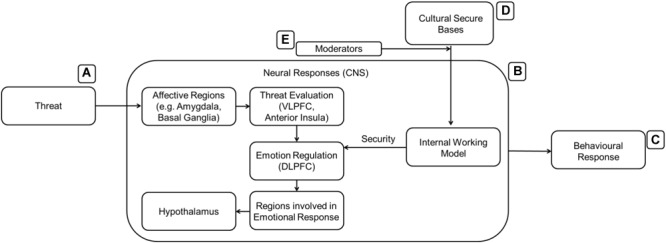
The IWM (cognitive framework, **B**) is activated by the presence of relevant cultural stimuli **(D)** and acts in tandem with the emotion regulation system to mitigate the threat experienced **(A)** before any behavioral response **(C)** is made. Coan (2016) as well as Vrticka and Vuilleumier (2012) both describe this top down executive control onto lower level affective processes. External factors **(E)** moderate the strength of the secure bases.

In order to integrate the cognitive and affective elements of CA, we further rely on the following model. Vrticka and Vuilleumier (2012; Figure 5) proposed a push-pull model between social approach and social avoidance (also known as the affective evaluation of the relationship) where secure attachment generally promotes social approach and vice versa. In their model, relatively basic and automatic affective evaluations are influenced by more elaborate cognitive control processes (such as emotion regulation and mental state representations). An example is Theory of Mind: the ability to understand the other person’s intentions allows individuals to attribute their actions differently and consequently adjust their behavior toward the other person. Subsequently, their behaviors also affect the way that individuals think, creating a bidirectional relationship between cognition and emotion. This suggests that the internal working model, being the cognitive part of the process, can now interact with the emotion regulation system – the affective part of the process. Although we are not currently able to detail the exact nature of this process, Figure 5 summarizes the moderation effect of the cognitive IWM elements onto the affective emotion regulation elements.

## Connecting the Body and the Brain

Major physiological theories of emotion (e.g., Somatic Marker Hypothesis; Damasio, 1996; Bechara et al., 2005) suggest that a significant part of emotional experience lies in the bodily response. Moreover, learning and memories are stronger when they are marked by bodily responses and are emotionally invested. SCR is one such bodily response that is commonly used as a biomarker of arousal to study emotional and threat processing. As described, the attachment system has been suggested to be an emotion regulation device (Vrticka and Vuilleumier, 2012) and insecure attachment styles have been associated with heightened SCR in response to stress (e.g., Sroufe and Waters, 1977b; Dozier and Kobak, 1992; Roisman et al., 2004; Gander and Buchheim, 2015). This means that learning cultural associations would be more successful if emotional responses are involved, not only during the acquisition of meaning (i.e., when one learns what culture is), but, as well, when these experiences and meanings are re-experienced throughout life. Interestingly, such associations would benefit from affective investment that involves peripheral bodily responses (“markers”) – such as heart rate or skin conductance.

Positive feelings of attachment facilitate the release of oxytocin into motivational circuits in the brain (Diamond, 2001; Campbell, 2010; Coan, 2010; Insel, 2010). Oxytocin and vasopressin are synthesized in large quantities in the hypothalamus (Gainer, 1994; Carter, 2003). The hypothalamus co-ordinates activity of physiological and behavioral systems during maternal or pair bonding (Kim et al., 2011) and is important in social soothing during neural threat (Carter, 2003; Coan et al., 2006). The hypothalamus (as part of the HPA axis) is also heavily involved in the regulation of autonomic nervous system responses. Oxytocin has also been associated with heightened aggression in response to perceived threat to others one shares strong social bonds with, such as offspring (Bosch et al., 2005). Notably, oxytocin has also been linked to intergroup bias among humans, suggesting that this neurobiological mechanism regulating pair bonding and attachment also contributes to broader group-level cohesion and defense (De Dreu, 2012).

## Toward a Neuroscience of Cultural Attachment

**FIGURE 6:**
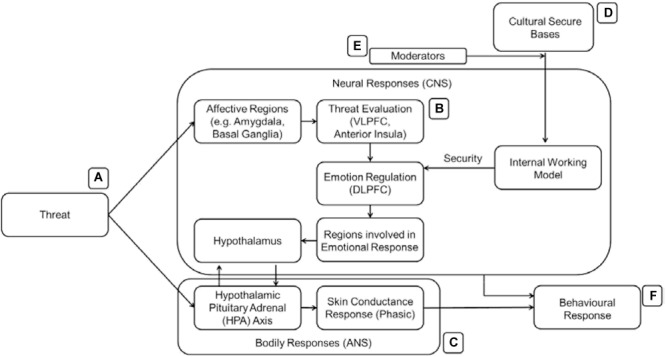
Full conceptual model. **(A)** Presence of threat leads to responses in the affective regions of the brain and ANS. **(B)** This is followed by threat evaluation and, if necessary, emotion regulation. The affective regions signal the threat evaluation regions which in turn signal emotion regulation regions if the threats are salient. If emotion regulation is successful, signals are fed back to affective regions and to other regions involved in emotional responses (e.g., motor, hypothalamus). **(C)** The HPA controls ANS responses which are typically subconscious and automatic responses to the environment. If emotion regulation is unsuccessful, SCR prepare the body for further responses. **(D)** Presence and availability of secure bases or the activation of the cognitive representation of secure bases is processed in the IWM before being passed on to aid emotion regulation. **(E)** External factors moderate the strength of the secure bases. **(F)** After the brain and body has assessed and processed the threat, a behavioral response is made, if necessary.

Following from Figure 5 and the previous section, we now incorporate the HPA axis into our conceptual model (Figure 6). To summarize, the full conceptual model is a mechanistic one which contains ideas from four established theories/models. The behavioral aspects of CA were combined with neurological findings associated with attachment as well as other basic emotional and cognitive functions (especially highlighting emotion regulation). The following four models were prioritized, as they have been developed to explain attachment mechanisms (the first three), whereas the fourth one is a generally accepted model explaining the relationship between bodily responses, brain mechanisms, and learning.

1. Coan (2016) model which describes dopamine release from reward processing regions to regions associated with emotion regulation during recall of previously salient positive experiences,
2. Kohn et al. (2014) model which refers to the emotion regulation process between affective and cognitive regions,
3. Vrticka and Vuilleumier (2012) model detailing cognitive and affective regions being moderated by different attachment styles,
4. Damasio and Bechara’s (Damasio, 1996; Bechara et al., 2005) Somatic Marker Hypothesis.

It should be noted that these models are based on more generic theories of the neurobiology of development, emotion and cognition (see the references above), and, as such we have not excluded major neuroscience theories.

In the full conceptual model (Figure 6), threatening stimuli are first processed in the affective regions of the brain and cause quick reactions from the HPA axis. Indeed, in a previous study (Yap et al., 2017) SCR were measured to examine the mitigation of threat in the presence of cultural symbols. The subliminal presentation of cultural stimuli reduced SCR – but only in the presence of a threatening stimulus. Following from Kohn et al. (2014), the affective regions in the brain then allows the salience of the threat to be evaluated and if the threat is identified as salient, then emotion regulatory processes take place. To aid in the success of the emotion regulation (or in this case threat mitigation), the IWM identifies the presence of rewarding socio-cultural cues and stimuli which results in dopamine release from reward processing regions to different parts of the prefrontal cortex involved in emotion regulation. The DLPFC (and other regions involved in emotion regulation) then signal the success (or failure) of the threat mitigation to the affective regions which then pass on the signal to the hypothalamus, motor regions, and other regions required to react to the threat. A behavioral response is then delivered.

Naturally, the conceptual model is not expanded to include all possible biological systems in play. Instead, we here focus on the cognitive and affective elements of attachment. When applying features of traditional AT to these different attachment targets, it is clear that there are some features compatible but there are also discrepancies that need to be reconciled.

In summary, the presence, availability, or activation of different secure bases in attachment (caregivers, romantic partners, cultural group, and the abstract mental representation of culture) is processed using the IWM (i.e., cognitive framework) to provide security and mitigate responses to threat. The success of the secure bases is moderated by the different factors such as need for cognitive closure or racial essentialism and social aspects, that will examined again at the discussion section.

## Discussion: Concepts, Models and Computational Culture Neuroscience

The conceptual model proposed highlights the magnitude of the entire body of research but also leads to more questions than answers. We discuss two general questions. Firstly, what is the relationship of CA with attachment as well as other similar concepts? Secondly, what are the neuro-computational mechanisms that could contribute to the development of CA?

### CA and Other Concepts

Starting with the theory of Attachment itself, it is currently unknown if all the features and major tenets of AT also apply to CA. However, there are definitely similarities behind the description of attachment and cultural processes to suggest that CA shares similarities with AT. The presence, availability, or activation of different secure bases in attachment (caregivers, romantic partners, cultural group, and the abstract mental representation of culture) is processed using the IWM (i.e., cognitive framework) to provide security and mitigate responses to threat. The pursuit of identifying the presence and nature of a cognitive framework is difficult without first establishing biological differences in threat responses due to CA. The effectiveness of culture as a secure base has been examined using behavioral experiments (Hong et al., 2013) and early results are encouraging. As converging, multi-modal evidence heavily reduces the possibility of spurious findings, psychophysiological and neuroscientific methods can be used to examine if the phenomenon occurs at a subconscious level.

Related to this, future research should be investigating the presence of a critical period of the acquisition of culture and the development of the CA bond. In the study of childhood attachment, age is a convenient proxy for brain development, thus using age as a proxy for a critical period is fitting. However, more understanding about cultural learning patterns is needed before the same can be said for CA. Young individuals who have not been exposed to different cultures should have a fairly stable model of their own culture. In addition, young adults are more capable of understanding cultural symbols as compared to younger children. More research might reveal transitional mechanisms in this area.

The relationship between basic social processes and cultural functions remains to be identified. For instance, group identity suggests that the membership of individuals in certain groups enhances their loyalty and positive feelings to that group and in contrast, increases their negative feelings toward members outside of the group and of other groups (Tajfel, 1974, 2010). Similar concepts are patriotism, which is the love and devotion for one’s country. Essentialism, and especially racial essentialism (see review Hong, 2015), could be outcomes of very strong CA mechanisms to the attachment figures. However, the exact relationship must be understood.

Another possible confound in our understanding of CA is the familiarity. Familiarity of a certain set of stimuli can bring about positive emotions which would provide a buffer against any negative ones. Familiarity is related with more emotional reactions such as nostalgia, the sentimental longing of one’s past, which can also induce positive emotions (Sedikides et al., 2004, 2008). Notice that our model includes a recall component that triggers the positive emotions. However, nostalgia and familiarity seem to cover less ground than attachment in that aspect.

From a more general point of view, CA highlights the importance of symbolism in cultural groups. The protective nature of cultural icons against threat emphasizes the importance of having identifiable symbols and the association of experiences within a cultural group to those symbols. Recent research in responses to perceived mixing of cultures have indeed suggested that people who are highly identified with their cultures may find mixing of foreign influences with symbols and representations of their own cultures especially aversive and distasteful (Cheon et al., 2016; Cheon, 2018). More narrow cultural groups also represent themselves with having clear and strong symbols. Some notable examples include luxury brands, sports teams, or even schools. Future research should examine possible attachment bonds to such cultural groups.

### Computational Culture Neuroscience

Both decision neuroscience and social neuroscience have been moving from descriptive approaches (predictivist models) to mechanistic approaches. This essentially involves incorporating computational descriptions of both behavior and neuronal responses. CA is particularly suited to be a testbed for introducing computational approaches to cultural phenomena, as we not only have paradigms that can help us elucidate brain mechanisms, but, as well, there seem to be strong relevance with existing theories of human neuroscience (described above).

Learning a culture and developing emotional bonds with its elements is certainly a life-long process where these meanings need to be (successfully or not) learned, unlearned and re-learned as the individual moves across space (for instance when somebody moves to a new culture) or across time (i.e., when culture evolves because of various technological, sociological or historical reasons). This, as well, is certainly a demanding task for the human brain, as the corpus of information to be accumulated is significant and, as well, many times ambiguous. “Being Chinese” or “Being Argentinian” – thus identity-would entail a very wide set of different behaviors, values, objects that they would need to be somehow summarized and represented.

How would the brain – and, actually, most computational systems – respond to such a challenge? The brain learns through many ways, but a principal function is the establishment of associations. Even at a basic neuronal level, synapses – the “junction” that permits the communication between neurons – are the basic learning units: when a neuronal cell is active enough to activate another neuronal cell (or systems of cells), then the two neuronal units will become “associated” – a process called Hebbian learning (Hebb, 1949). This seemingly simple process is actually computationally adequate – when a large number of neurons is involved – to not only represent a vast amount of information, but, as well, to allow the brain to dynamically respond and adapt to changes. Such associative process can explain the emergence of functions that support social functions, such as the mirror neurons. Computationally, associative processes need to use predictions, and prediction error computations; activity of the dopaminergic system behaves as if it carries out such computations. Temporal precedence is another computationally necessary function: a neuron needs to fire before the next neuron – thus the colloquial dictum “neurons that fire together wire together” is not exact.

Associative mechanisms are the basis of implicit learning and support long-term memory functions. Associations can be established across a wide range of stimuli. The image of MacDonald’s Arcs does not itself satisfy the need of hunger, but the image has been associated with receiving (delicious or not) food. Processes similar to what happens at the neuronal level follow: the image temporally precedes the food; the consumer has to make predictions (i.e., have “expectations” in the culture science terminology) and compute prediction errors (i.e., evaluate the outcome as compared to expectations). Importantly, MacDonald’s logo does not only summarize the burger somebody eats, but actually the sum of the whole experience – that is the bodily responses, the emotions (for instance feeling happy, contend, secure), thoughts, social interactions. With no doubt, these experiences and expectations differ from individual to individual, but the point is that the symbol conveniently represents a rather complex set of meanings, memories and internal states. This is very useful, not only because the representation of the social environment becomes less expensive in computational terms, but, critically, because such complex schemas can be shared and communicated with others.

These processes can and have been described based on a central computation – i.e., the computation of prediction errors. This simple computational element can allow for the formation of generative and forward-looking models of the world, which can be extremely abstract (Clark, 2016, 2017) As such, the computational framework of reward learning, as described by recent advances in decision neuroscience might be of particular relevance for the understanding of the development and stabilization of CA. Indeed, closely related theories such as niche construction and evolutionary approaches have been for some time considering computational approaches, including the free energy principle (Laland et al., 2015; Constant et al., 2018). The integration of ideas from culture theory, anthropology, neuroscience, and artificial intelligence could lead to a much deeper understanding of the processes that underlie how humans and cultures co-evolve.

## Author Contributions

W-JY and GC wrote the first versions of the manuscript, subsequently all authors added text and made changes to finalize the manuscript. All authors discussed and conceptualized the manuscript.

## Conflict of Interest Statement

The authors declare that the research was conducted in the absence of any commercial or financial relationships that could be construed as a potential conflict of interest.

## References

[B1] Ainsworth M. D. (1963). “The development of infant-mother interaction among the Ganda,” in *Determinants of Infant Behavior* ed. Foss B. M. (New York, NY: Wiley) 67–112.

[B2] Ainsworth M. D. Wittig B. A. (1969). “Attachment and exploratory behaviour in 1-year olds in a strange situation,” in *Determinants of Infant Behaviour* ed. Foss B. M. (London: Methuen).

[B3] Ainsworth M. D. S. Blehar M. C. Waters E. Wall S. (1978). *Patterns of Attachment.* Hillsdale, NJ: Lawrence Erlbaum Associates, Inc.

[B4] Ainsworth M. S. Bowlby J. (1991). An ethological approach to personality development. *Am. Psychol.* 46 333–341. 10.1037//0003-066x.46.4.333

[B5] Amodio D. M. Frith C. D. (2006). Meeting of minds: the medial frontal cortex and social cognition. *Nat. Rev. Neurosci.* 7 268–277. 10.1038/nrn1884 16552413

[B6] Aron A. Fisher H. Mashek D. J. Strong G. Li H. Brown L. L. (2005). Reward, motivation, and emotion systems associated with early-stage intense romantic love. *J. Neurophysiol.* 94 327–337. 10.1152/jn.00838.2004 15928068

[B7] Associated Press (2018). *Greek Strike to Close Acropolis, other Key Sites on Oct. 11.* New York, NY: Associated Press.

[B8] Banse R. (1999). Automatic evaluation of self and significant others: affective priming in close relationships. *J. Soc. Pers. Relationsh.* 16 803–821. 10.1177/0265407599166007

[B9] Banse R. (2001). Affective priming with liked and disliked persons: prime visibility determines congruency and incongruency effects. *Cogn. Emot.* 15 501–520. 10.1080/0269993004200213

[B10] Bargh J. A. Chartrand T. L. (2000). “The mind in the middle: a practical guide to priming and automaticity research,” in *Handbook of Research Methods in Social and Personality Psychology* eds Reis H. T. Judd C. M. (New York, NY: Cambridge University Press) 253–285.

[B11] Bartels A. Zeki S. (2000). The neural basis of romantic love. *Neuroreport* 11 3829–3834. 10.1097/00001756-200011270-00046 11117499

[B12] Bartels A. Zeki S. (2004). The neural correlates of maternal and romantic love. *Neuroimage* 21 1155–1166. 10.1016/j.neuroimage.2003.11.003 15006682

[B13] Bechara A. Damasio H. Tranel D. Damasio A. R. (2005). The Iowa Gambling Task and the somatic marker hypothesis: some questions and answers. *Trends Cogn. Sci.* 9 159–162. 10.1016/j.tics.2005.02.002 15808493

[B14] Bechtel W. Richardson R. C. (1993). *Discovering Complexity: Decomposition and Localization as Scientific Research Strategies.* Princeton, NJ: Princeton University Press.

[B15] Becker G. S. (2013). *The Economic Approach to Human Behavior.* Chicago, IL: University of Chicago Press.

[B16] Beckes L. Coan J. A. (2015). “The distress-relief dynamic in attachment bonding,” in *Bases of Adult Attachment* eds Zayas V. Hazan C. (New York, NY: Springer) 11–33. 10.1007/978-1-4614-9622-9_2

[B17] Beckes L. Simpson J. A. Erickson A. (2010). Of snakes and succor: learning secure attachment associations with novel faces via negative stimulus pairings. *Psychol. Sci.* 21 721–728. 10.1177/0956797610368061 20483852

[B18] Belsky J. Spritz B. Crnic K. (1996). Infant attachment security and affective-cognitive information processing at age 3. *Psychol. Sci.* 7 111–114. 10.1111/j.1467-9280.1996.tb00339.x

[B19] Belsky J. A. Y. Fearon R. P. (2002). Infant-mother attachment security, con-textual risk, and early development: a moderational analysis. *Dev. Psychopathol.* 14 293–310. 10.1017/s0954579402002067 12030693

[B20] Bosch O. J. Meddle S. L. Beiderbeck D. I. Douglas A. J. Neumann I. D. (2005). Brain oxytocin correlates with maternal aggression: link to anxiety. *J. Neurosci.* 25 6807–6815. 10.1523/jneurosci.1342-05.2005 16033890 PMC6725361

[B21] Bowlby J. (1969). *Attachment and Loss: Attachment* Vol. 1 New York, NY: Basic Books

[B22] Bowlby J. (1973). *Attachment and Loss: Separation, Anxiety and Anger* Vol. 2 New York, NY: Basic Books

[B23] Bowlby J. (1980). *Attachment and Loss: Loss, Sadness and Depression* Vol. 3 New York, NY: Basic Books

[B24] Bowlby J. (1982). Attachment and loss: retrospect and prospect. *Am. J. Orthopsychiatry* 52 664–678. 10.1111/j.1939-0025.1982.tb01456.x 7148988

[B25] Bowlby J. (1988). Developmental psychiatry comes of age. *Am. J. Psychiatry* 145 1–10. 10.1176/ajp.145.1.1 3276225

[B26] Bowlby J. (2008). *A Secure Base: Parent-Child Attachment and Healthy Human Development.* New York, NY: Basic Books.

[B27] Brasted P. Bussey T. Murray E. Wise S. (2003). Role of the hippocampal system in associative learning beyond the spatial domain. *Brain* 126 1202–1223. 10.1093/brain/awg103 12690059

[B28] Bretherton I. (1985). Attachment theory: retrospect and prospect. *Monogr. Soc. Res. Child Dev.* 50 3–35.

[B29] Bretherton I. (1990). “Open communication and internal working models: their role in the development of attachment relationships,” in *Current theory and Research in Motivation, Nebraska Symposium on Motivation, 1988: Socioemotional Development* Vol. 36 ed. Thompson R. A. (Lincoln, NE: University of Nebraska Press) 57–113. 3078938

[B30] Bretherton I. (1993). “From dialogue to internal working models: the co-construction of self in relationships,” in *Minnesota Symposium on Child Psychology: Memory and Affect in Development* Vol. 26 ed. Nelson C. A. (Hillsdale, NJ: Erlbaum) 237–264.

[B31] Bretherton I. Munholland K. A. (2008). “Internal working models in attachment relationships: elaborating a central construct in attachment theory,” in *Handbook of Attachment* 2nd Edn eds Cassidy J. Shaver P. (New York, NY: Guilford Press).

[B32] Briley D. (2008). Biculturals, conformity motives and decision making. *Int. J. Psychol.* 43 184–184.

[B33] Briley D. A. Morris M. W. Simonson I. (2000). Reasons as carriers of culture: dynamic versus dispositional models of cultural influence on decision making. *J. Consum. Res.* 27 157–178. 10.1086/314318

[B34] Briley D. A. Morris M. W. Simonson I. (2005). Cultural chameleons: biculturals, conformity motives, and decision making. *J. Consum. Psychol.* 15 351–362. 10.1207/s15327663jcp1504_9

[B35] Briley D. A. Wyer R. S. Jr. (2001). Transitory determinants of values and decisions: the utility (or nonutility) of individualism and collectivism in understanding cultural differences. *Soc. Cogn.* 19 197–227. 10.1521/soco.19.3.197.21474

[B36] Buchheim A. George C. Gndel H. Viviani R. (2017). Neuroscience of human attachment. *Front. Hum. Neurosci.* 11:136. 10.3389/fnhum.2017.00136 28392762 PMC5364168

[B37] Cahill L. Prins B. Weber M. McGaugh J. L. (1994). β-Adrenergic activation and memory for emotional events. *Nature* 371 702–704. 10.1038/371702a0 7935815

[B38] Campbell A. (2010). Oxytocin and human social behavior. *Pers. Soc. Psychol. Rev.* 14 281–295. 10.1177/1088868310363594 20435805

[B39] Canterberry M. Gillath O. (2013). Neural evidence for a multifaceted model of attachment security. *Int. J. Psychophysiol.* 88 232–240. 10.1016/j.ijpsycho.2012.08.013 22940284

[B40] Carter C. S. (2003). Developmental consequences of oxytocin. *Physiol. Behav.* 79 383–397. 10.1016/s0031-9384(03)00151-3 12954433

[B41] Cassidy J. (1988). Child-mother attachment and the self in six-year-olds. *Child Dev.* 59 121–134. 10.1111/j.1467-8624.1988.tb03200.x 3342707

[B42] Cassidy J. Kirsh S. J. Scolton K. L. Parke R. D. (1996). Attachment and representations of peer relationships. *Dev. Psychol.* 32 892–904. 10.1037//0012-1649.32.5.892

[B43] Cassidy J. Marvin R. S. MacArthur Working Group (1992). *Attachment Organization in Preschool Children: Procedures and Coding Manual.* Charlottesville, VA: University of Virginia 125–131.

[B44] Cassidy J. Shaver P. (2016). *Handbook of Attachment: Theory, Research, and Clinical Applications.* New York, NY: Guilford Press.

[B45] Cassidy J. Ziv Y. Mehta T. G. Feeney B. C. (2003). Feedback seeking in children and adolescents: associations with self-perceptions, attachment representations, and depression. *Child Dev.* 74 612–628. 10.1111/1467-8624.7402019 12705576

[B46] Chao M. M. Kung F. Y. Yao D. J. (2015). Understanding the divergent effects of multicultural exposure. *Int. J. Intercult. Relat.* 47 78–88. 10.1016/j.ijintrel.2015.03.032

[B47] Chen H. Ng S. Rao A. R. (2005). Cultural differences in consumer impatience. *J. Mark. Res.* 42 291–301. 10.1509/jmkr.2005.42.3.291

[B48] Cheon B. K. (2018). The diversity of cultural diversity: psychological consequences of different patterns of intercultural contact and mixing. *Asian J. Soc. Psychol.* 22 93–105. 10.1111/ajsp.12321

[B49] Cheon B. K. Christopoulos G. I. Hong Y. (2016). Disgust associated with culture mixing: why and who? *J. Cross Cult. Psychol.* 47 1268–1285. 10.1177/0022022116667845

[B50] Chiao J. Y. Ambady N. (2007). “Cultural neuroscience: parsing universality and diversity across levels of analysis,” in *Handbook of Cultural Psychology* eds Kitayama S. Cohen D. (New York, NY: Guilford Press).

[B51] Chiao J. Y. Harada T. Komeda H. Li Z. Mano Y. Saito D. (2010). Dynamic cultural influences on neural representations of the self. *J. Cogn. Neurosci.* 22 1–11. 10.1162/jocn.2009.21192 19199421

[B52] Christopoulos G. I. Tobler P. N. (2016). “Culture as a response to uncertainty: foundations of computational cultural neuroscience,” in *The Oxford Handbook of Cultural Neuroscience* eds Chiao J. Y. Li S.-C. Seligman R. Turner R. (New York, NY: Oxford University Press) 81–106.

[B53] Clark A. (2016). *Surfing Uncertainty: Prediction, Action, and the Embodied Mind.* Oxford: Oxford University Press 10.1093/acprof:oso/9780190217013.001.0001

[B54] Clark A. (2017). A nice surprise? Predictive processing and the active pursuit of novelty. *Phenomenol. Cogn. Sci.* 17 521–534. 10.1007/s11097-017-9525-z

[B55] Coan J. (2016). “Attachment and neuroscience,” in *Handbook of Attachment* 3rd Edn eds Cassidy J. Shaver P. (New York, NY: Guilford Press).

[B56] Coan J. A. (2008). “Toward a neuroscience of attachment,” in *Handbook of Attachment: Theory, Research, and Clinical Applications* Vol. 2 eds Cassidy J. Shaver P. R. (New York, NY: Guilford Press) 241–265.

[B57] Coan J. A. (2010). Adult attachment and the brain. *J. Soc. Pers. Relationsh.* 27 210–217.

[B58] Coan J. A. Sbarra D. A. (2015). Social baseline theory: the social regulation of risk and effort. *Curr. Opin. Psychol.* 1 87–91. 10.1016/j.copsyc.2014.12.021 25825706 PMC4375548

[B59] Coan J. A. Schaefer H. S. Davidson R. J. (2006). Lending a hand: social regulation of the neural response to threat. *Psychol. Sci.* 17 1032–1039. 10.1111/j.1467-9280.2006.01832.x 17201784

[B60] Constant A. Ramstead M. J. Veissiere S. P. Campbell J. O. Friston K. J. (2018). A variational approach to niche construction. *J. R. Soc. Interface* 15:20170685. 10.1098/rsif.2017.0685 PMC5938575 29643221

[B61] Craver C. F. (2007). *Explaining the Brain: Mechanisms and the Mosaic Unity of Neuroscience.* Oxford: Oxford University Press.

[B62] Damasio A. R. (1996). The somatic marker hypothesis and the possible functions of the prefrontal cortex. *Philos. Trans. R. Soc. Lond. B Biol. Sci.* 351 1413–1420. 10.1098/rstb.1996.0125 8941953

[B63] Davidovitz R. Mikulincer M. Shaver P. R. Izsak R. Popper M. (2007). Leaders as attachment figures: leaders’ attachment orientations pre-dict leadership-related mental representations and followers’ performance and mental health. *J. Pers. Soc. Psychol.* 93 632–650. 10.1037/0022-3514.93.4.632 17892336

[B64] Dayan P. Abbott L. F. (2001). *Theoretical Neuroscience: Computational and Mathematical Modeling of Neural Systems.* Cambridge, MA: MIT Press.

[B65] De Berker A. O. Rutledge R. B. Mathys C. Marshall L. Cross G. F. Dolan R. J. (2016). Computations of uncertainty mediate acute stress responses in humans. *Nat. Commun.* 7:10996. 27020312 10.1038/ncomms10996 PMC4820542

[B66] De Dreu C. K. (2012). Oxytocin modulates cooperation within and competition between groups: an integrative review and research agenda. *Horm. Behav.* 61 419–428. 10.1016/j.yhbeh.2011.12.009 22227278

[B67] Denham S. Mason T. Caverly S. Schmidt M. Hackney R. Caswell C. (2001). Preschoolers at play: co-socialisers of emotional and social competence. *Int. J. Behav. Dev.* 25 290–301. 10.1080/016502501143000067

[B68] Denham S. A. Blair K. Schmidt M. DeMulder E. (2002). Compromised emotional competence: seeds of violence sown early? *Am. J. Orthopsychiatry* 72 70–82. 10.1037//0002-9432.72.1.70 14964596

[B69] Depue R. A. Morrone-Strupinsky J. V. (2005). A neurobehavioral model of affiliative bonding: implications for conceptualizing a human trait of affiliation. *Behav. Brain Sci.* 28 313–349. 16209725 10.1017/S0140525X05000063

[B70] Diamond L. M. (2001). Contributions of psychophysiology to research on adult attachment: review and recommendations. *Pers. Soc. Psychol. Rev.* 5 276–295. 10.1207/s15327957pspr0504_1

[B71] Diamond L. M. Hicks A. M. Otter-Henderson K. (2006). Physiological evidence for repressive coping among avoidantly attached adults. *J. Soc. Pers. Relationsh.* 23 205–229. 10.1177/0265407506062470

[B72] Diener A. C. (2002). National territory and the reconstruction of history in Kazakhstan. *Eurasian Geogr. Econ.* 43 632–650. 10.2747/1538-7216.43.8.632

[B73] Dienske H. Metz J. (1977). Mother-infant body contact in macaques: a time interval analysis. *Biol. Behav.* 2 3–37.

[B74] Dijksterhuis A. Bargh J. A. (2001). The perception-behavior expressway: automatic effects of social perception on social behavior. *Adv. Exp. Soc. Psychol.* 33 1–40. 10.1016/s0065-2601(01)80003-4

[B75] Doyle A. B. Markiewicz D. Brendgen M. Lieberman M. Voss K. (2000). Child attachment security and self-concept: associations with mother and father attachment style and marital quality. *Merrill Palmer Q.* 46 514–539.

[B76] Dozier M. Kobak R. R. (1992). Psychophysiology in attachment interviews: converging evidence for deactivating strategies. *Child Dev.* 63 1473–1480. 10.1111/j.1467-8624.1992.tb01708.x 1446563

[B77] Dunn J. (1993). *Young Children’s Close Relationships: Beyond Attachment.* Thousand Oaks, CA: Sage Publications, Inc.

[B78] Dykas M. J. Cassidy J. (2011). Attachment and the processing of social information across the life span: theory and evidence. *Psychol. Bull.* 137 19–46. 10.1037/a0021367 21219056

[B79] Egeland B. Kalkoske M. Gottesman N. Erickson M. F. (1990). Preschool behavior problems: stability and factors accounting for change. *J. Child Psychol. Psychiatry* 31 891–909. 10.1111/j.1469-7610.1990.tb00832.x 2246340

[B80] Eisenberg M. Shmuelof L. Vaadia E. Zohary E. (2011). The representation of visual and motor aspects of reaching movements in the human motor cortex. *J. Neurosci.* 31 12377–12384. 10.1523/jneurosci.0824-11.2011 21865480 PMC6623232

[B81] Elaine Perunovic W. Q. Heller D. Rafaeli E. (2007). Within-person changes in the structure of emotion: the role of cultural identification and language. *Psychol. Sci.* 18 607–613. 10.1111/j.1467-9280.2007.01947.x 17614869

[B82] Ellenbogen M. A. Schwartzman A. E. Stewart J. Walker C.-D. (2006). Automatic and effortful emotional information processing regulates different aspects of the stress response. *Psychoneuroendocrinology* 31 373–387. 10.1016/j.psyneuen.2005.09.001 16289608

[B83] Feeney B. C. (2004). A secure base: responsive support of goal strivings and exploration in adult intimate relationships. *J. Pers. Soc. Psychol.* 87 631–648. 10.1037/0022-3514.87.5.631 15535776

[B84] Fox N. Hane A. (2008). “Studying the biology of human attachment,” in *Handbook of Attachment: Theory, Research, and Clinical Applications* Vol. 2 eds Cassidy J. Shaver P. R. (New York, NY: Guilford Press) 217–240.

[B85] Fu J. H.-Y. Chiu C.-Y. Morris M. W. Young M. J. (2007a). Spontaneous inferences from cultural cues: varying responses of cultural insiders and outsiders. *J. Cross Cult. Psychol.* 38 58–75. 10.1177/0022022106295443

[B86] Fu J. H.-Y. Morris M. W. Lee S.-L. Chao M. Chiu C.-Y. Hong Y. (2007b). Epistemic motives and cultural conformity: need for closure, culture, and context as determinants of conflict judgments. *J. Pers. Soc. Psychol.* 92 191–207. 10.1037/0022-3514.92.2.191 17279845

[B87] Fuster J. M. (1997). Network memory. *Trends Neurosci.* 20 451–459. 9347612 10.1016/s0166-2236(97)01128-4

[B88] Fu J. H.-Y. Morris M. W. Hong Y. Y. (2015). A transformative taste of home: home culture primes foster expatriates’ adjustment through bolstering relational security. *J. Exp. Soc. Psychol.* 59 24–31. 10.1016/j.jesp.2015.02.004

[B89] Gainer H. (1994). “Cellular and molecular biology of oxytocin and vasopressin,” in *The Physiology of Reproduction* eds Knobil E. Neill J. D. (New York, NY: Raven Press) 1099–1129.

[B90] Gander M. Buchheim A. (2015). Attachment classification, psychophysiology and frontal EEG asymmetry across the lifespan: a review. *Front. Hum. Neurosci.* 9:79. 10.3389/fnhum.2015.00079 25745393 PMC4333768

[B91] Gee D. G. Gabard-Durnam L. J. Flannery J. Goff B. Humphreys K. L. Telzer E. H. (2013). Early developmental emergence of human amygdala-prefrontal connectivity after maternal deprivation. *Proc. Natl. Acad. Sci. U.S.A.* 110 15638–15643. 10.1073/pnas.1307893110 24019460 PMC3785723

[B92] George C. West M. (2001). The development and preliminary validation of a new measure of adult attachment: the adult attachment projective. *Attach. Hum. Dev.* 3 30–61. 10.1080/14616730010024771 11708383

[B93] Glucklich A. (2009). Dying for heaven: holy pleasure and suicide bombers-why the best qualities of religion are also its most dangerous. *Relig. Stud. Rev.* 37:26.

[B94] Granqvist P. Ljungdahl C. Dickie J. R. (2007). God is nowhere, God is now here: attachment activation, security of attachment, and God’s perceived closeness among 57-year-old children from religious and non-religious homes. *Attach. Hum. Dev.* 9 55–71. 10.1080/14616730601151458 17364482

[B95] Greenberg M. T. Marvin R. S. (1982). Reactions of preschool children to an adult stranger: a behavioral systems approach. *Child Dev.* 53 481–490. 10.1111/j.1467-8624.1982.tb01338.x

[B96] Grieve P. G. Hogg M. A. (1999). Subjective uncertainty and intergroup dis-crimination in the minimal group situation. *Pers. Soc. Psychol. Bull.* 25 926–940. 10.1177/01461672992511002 9738312

[B97] Hamann S. B. Ely T. D. Grafton S. T. Kilts C. D. (1999). Amygdala activity related to enhanced memory for pleasant and aversive stimuli. *Nat. Neurosci.* 2 289–293. 10.1038/6404 10195224

[B98] Harlow H. F. Harlow M. K. (1965). The affectional systems. *Behav. Nonhum. Primates* 2 287–334. 10.1016/b978-1-4832-2821-1.50008-2

[B99] Harter S. Pike R. (1984). The pictorial scale of perceived competence and social acceptance for young children. *Child Dev.* 55 1969–1982. 10.1111/j.1467-8624.1984.tb03895.x 6525886

[B100] Hazan C. Shaver P. (1987). Romantic love conceptualized as an attachment process. *J. Pers. Soc. Psychol.* 52 511–524. 10.1037//0022-3514.52.3.511 3572722

[B101] Hebb D. O. (1949). *The Organization of Behavior.* New York, NY: Wiley and Sons.

[B102] Higgins E. T. (1996). “Knowledge activation: accessibility, applicability, and salience,” in *Social Psychology: Handbook of Basic Principles* eds Higgins E. T. Kruglanski A. W. (New York, NY: Guilford Press) 133–168.

[B103] Hofer M. A. (2006). Psychobiological roots of early attachment. *Curr. Dir. Psychol. Sci.* 15 84–88. 10.1111/j.0963-7214.2006.00412.x 803142

[B104] Hofstede G. (2011). Dimensionalizing cultures: the Hofstede model in context. *Online Read. Psychol. Cult.* 2 10.9707/2307-0919.1014

[B105] Hong Y. (2009). “A dynamic constructivist approach to culture,” in *Problems and Solutions in Cross-Cultural Theory, Research and Application* eds Wyer R. S. Chiu C. Y. Hong Y. Y. (New York, NY: Psychology Press) 2–24.

[B106] Hong Y. (2015). “Lay theory of race,” in *Encyclopedia of Intercultural Competence* Vol. 2 ed. Bennett J. M. (Thousand Oaks, CA: Sage) 584–586.

[B107] Hong Y. (2017). “Cultural attachment theory,” in *The International Encyclopedia of Intercultural Communication* ed. Kim Y. Y. (Hoboken, NJ: John Wiley) 1–10.

[B108] Hong Y. Benet-Martinez V. Chiu C.-Y. Morris M. W. (2003). Boundaries of cultural influence: construct activation as a mechanism for cultural differences in social perception. *J. Cross Cult. Psychol.* 34 453–464. 10.1177/0022022103034004005

[B109] Hong Y.-Y. Chiu C.-Y. Kung T. M. (1997). Bringing culture out in front: effects of cultural meaning system activation on social cognition. *Progress Asian Soc. Psychol.* 1 135–146.

[B110] Hong Y. Fang Y. Yang Y. Phua D. Y. (2013). Cultural attachment: a new theory and method to understand cross-cultural competence. *J. Cross Cult. Psychol.* 44 1024–1044. 10.1177/0022022113480039

[B111] Hong Y. Khei M. (2014). “Dynamic multiculturalism: the interplay of socio-cognitive, neural and genetic mechanisms,” in *The Oxford Handbook of Multicultural Identity* eds Benet-Martinez V. Hong Y. (New York, NY: Oxford University Press) 11–34.

[B112] Hong Y. Morris M. W. Chiu C.-Y. Benet-Martinez V. (2000). Multicultural minds: a dynamic constructivist approach to culture and cognition. *Am. Psychol.* 55 709–720. 10.1037//0003-066x.55.7.709 10916861

[B113] Hong Y. Roisman G. I. Chen J. (2006). “A model of cultural attachment: a new approach for studying bicultural experience: measurement and development,” in *Acculturation and Parent-Child Relationships: Measurement and Development* eds Bornstein M. H. Cote L. R. (New York, NY: Erlbaum) 135–170.

[B114] Hong Y.-Y. Zhan S. Morris M. W. Benet-Martínez V. (2016). Multicultural identity processes. *Curr. Opin. Psychol.* 8 49–53. 10.1016/j.copsyc.2015.09.020 29506802

[B115] Insel T. R. (2010). The challenge of translation in social neuroscience: a review of oxytocin, vasopressin, and affiliative behavior. *Neuron* 65 768–779. 10.1016/j.neuron.2010.03.005 20346754 PMC2847497

[B116] Kanske P. Heissler J. Schnfelder S. Bongers A. Wessa M. (2010). How to regulate emotion? Neural networks for reappraisal and distraction. *Cereb. Cortex* 21 1379–1388. 10.1093/cercor/bhq216 21041200

[B117] Keefer L. A. (2016). Is there anybody out there? *J. Individ. Dif.* 37 231–238.

[B118] Keefer L. A. Landau M. J. Sullivan D. (2014). Non-human support: broadening the scope of attachment theory. *Soc. Pers. Psychol. Compass* 8 524–535. 10.1111/spc3.12129

[B119] Kennedy P. J. Shapiro M. L. (2004). Retrieving memories via internal context requires the hippocampus. *J. Neurosci.* 24 6979–6985. 10.1523/jneurosci.1388-04.2004 15295033 PMC6729595

[B120] Kerns K. A. Abraham M. M. Schlegelmilch A. Morgan T. A. (2007). Mother-child attachment in later middle childhood: assessment approaches and associations with mood and emotion regulation. *Attach. Hum. Dev.* 9 33–53. 10.1080/14616730601151441 17364481

[B121] Kerns K. A. Klepac L. Cole A. (1996). Peer relationships and pre-adolescents’ perceptions of security in the child-mother relationship. *Dev. Psychol.* 32 457–466. 10.1037//0012-1649.32.3.457

[B122] Khan J. (2012). *The Quest for Desi, Halal Food in America*. Available at: https://www.voanews.com/a/the-quest-for-desi-halal-food-inamerica/4008864.html (accessed August 1 2018).

[B123] Kim P. Feldman R. Mayes L. C. Eicher V. Thompson N. Leckman J. F. (2011). Breastfeeding, brain activation to own infant cry, and maternal sensitivity. *J. Child Psychol. Psychiatry* 52 907–915. 10.1111/j.1469-7610.2011.02406.x 21501165 PMC3134570

[B124] Kirkpatrick L. A. (2005). *Attachment, Evolution, and the Psychology of Religion.* New York, NY: Guilford Press.

[B125] Kluckhohn C. (1954). Southwestern studies of culture and personality. *Am. Anthropol.* 56 685–697. 10.1525/aa.1954.56.4.02a00280

[B126] Kodirov S. A. (2012). “The role of norepinephrine in amygdala dependent fear learning and memory,” in *The Amygdala - A Discrete Multitasking Manager* ed. Ferry B. (London: InTechOpen) 121–140.

[B127] Kohn N. Eickhoff S. B. Scheller M. Laird A. R. Fox P. T. Habel U. (2014). Neural network of cognitive emotion regulation an ALE meta-analysis and MACM analysis. *Neuroimage* 87 345–355. 10.1016/j.neuroimage.2013.11.001 24220041 PMC4801480

[B128] Krawczyk D. C. (2002). Contributions of the prefrontal cortex to the neural basis of human decision making. *Neurosci. Biobehav. Rev.* 26 631–664. 10.1016/s0149-7634(02)00021-0 12479840

[B129] Labek K. Viviani R. Gizewski E. R. Verius M. Buchheim A. (2016). Neural correlates of the appraisal of attachment scenes in healthy controls and social cognition: an fMRI study. *Front. Hum. Neurosci.* 10:345. 10.3389/fnhum.2016.00345 27458363 PMC4932100

[B130] Laible D. J. Thompson R. A. (1998). Attachment and emotional understanding in preschool children. *Dev. Psychol.* 34 1038–1045. 10.1037/0012-1649.34.5.1038 9779749

[B131] Laird J. D. Strout S. (2007). “Emotional behaviors as emotional stimuli,” in *Hand-Book of Emotion Elicitation and Assessment* eds Coan J. A. Allen J. J. B. (New York, NY: Oxford University Press) 54–64.

[B132] Laland K. N. Uller T. Feldman M. W. Sterelny K. Müller G. B. Moczek A. (2015). The extended evolutionary synthesis: its structure, assumptions and predictions. *Proc. R. Soc. B Biol. Sci.* 282:20151019. 10.1098/rspb.2015.1019 26246559 PMC4632619

[B133] LeVine R. A. (2010). “Anthropological foundations of cultural psychology,” in *Handbook of Cultural Psychology* eds Kitayama S. Cohen D. (New York, NY: Guilford Press).

[B134] Lewicka M. (2011). Place attachment: how far have we come in the last 40 years? *J. Environ. Psychol.* 31 207–230. 10.1016/j.jenvp.2010.10.001

[B135] Liang K. C. Chen L. L. Huang T.-E. (1995). The role of amygdala norepinephrine in memory formation: involvement in the memory enhancing effect of peripheral epinephrine. *Chin. J. Physiol.* 38 81–91. 8697902

[B136] Machamer P. Darden L. Craver C. F. (2000). Thinking about mechanisms. *Philos. Sci.* 67 1–25.

[B137] Magai C. (2008). “Attachment in middle and later life,” in *Handbook of Attachment: Theory, Research, and Clinical Applications* 2nd Edn eds Cassidy J. Shaver P. R. (New York, NY: Guilford Press) 532–551.

[B138] Main M. Kaplan N. Cassidy J. (1985). Security in infancy, childhood, and adulthood: a move to the level of representation. *Monogr. Soc. Res. Child Dev.* 50 66–104.

[B139] Mikulincer M. (1998). Attachment working models and the sense of trust: an exploration of interaction goals and affect regulation. *J. Pers. Soc. Psychol.* 74 1209–1224. 10.1037//0022-3514.74.5.1209

[B140] Mikulincer M. Florian V. (1995). Appraisal of and coping with a real-life stressful situation: the contribution of attachment styles. *Pers. Soc. Psychol. Bull.* 21 406–414. 10.1177/0146167295214011

[B141] Mikulincer M. Shaver P. R. (2011). “Attachment, anger, and aggression,” in *Human Aggression and Violence: Causes, Manifestations, and Consequences* eds Shaver P. R. Mikulincer M. (Washington, DC: American Psychological Association) 241–257.

[B142] Mikulincer M. Florian V. Cowan P. A. Cowan C. P. (2002a). Attachment security in couple relationships: a systemic model and its implications for family dynamics. *Fam. Process* 41 405–434. 10.1111/j.1545-5300.2002.41309.x 12395567

[B143] Mikulincer M. Gillath O. Shaver P. R. (2002b). Activation of the attachment system in adulthood: threat-related primes increase the accessibility of mental representations of attachment figures. *J. Pers. Soc. Psychol.* 83 881–895. 10.1037//0022-3514.83.4.881 12374442

[B144] Mikulincer M. Gillath O. Halevy V. Avihou N. Avidan S. Eshkoli N. (2001a). Attachment theory and reactions to others’ needs: evidence that activation of the sense of attachment security promotes empathic responses. *J. Pers. Soc. Psychol.* 81 1205–1224. 10.1037/0022-3514.81.6.1205 11761318

[B145] Mikulincer M. Hirschberger G. Nachmias O. Gillath O. (2001b). The affective component of the secure base schema: affective priming with representations of attachment security. *J. Pers. Soc. Psychol.* 81 305–321. 10.1037//0022-3514.81.2.305 11519934

[B146] Mikulincer M. Shaver P. R. (2001). Attachment theory and intergroup bias: evidence that priming the secure base schema attenuates negative reactions to out-groups. *J. Pers. Soc. Psychol.* 81 97–115. 10.1037//0022-3514.81.1.97 11474729

[B147] Mikulincer M. Shaver P. R. (2003). The attachment behavioral system in adulthood: activation, psychodynamics, and interpersonal processes. *Adv. Exp. Soc. Psychol.* 35 53–152. 10.1016/s0065-2601(03)01002-5

[B148] Mikulincer M. Shaver P. R. (2004). “Security-based self-representations in adulthood,” in *Adult Attachment: Theory, Research, and Clinical Implications* eds Rholes W. S. Simpson J. A. (New York, NY: Guilford Publications) 159–195.

[B149] Mikulincer M. Shaver P. R. (2007a). *Attachment in Adulthood: Structure, Dynamics, and Change.* New York, NY: Guilford Press.

[B150] Mikulincer M. Shaver P. R. (2007b). Reflections on security dynamics: core constructs, psychological mechanisms, relational contexts, and the need for an integrative theory. *Psychol. Inq.* 18 197–209. 10.1080/10478400701512893

[B151] Mikulincer M. Shaver P. R. Sapir-Lavid Y. Avihou-Kanza N. (2009). What’s inside the minds of securely and insecurely attached people? The secure-base script and its associations with attachment-style dimensions. *J. Pers. Soc. Psychol.* 97 615–633. 10.1037/a0015649 19785482

[B152] Milad M. R. Quinn B. T. Pitman R. K. Orr S. P. Fischl B. Rauch S. L. (2005). Thickness of ventromedial prefrontal cortex in humans is correlated with extinction memory. *Proc. Natl. Acad. Sci. U.S.A.* 102 10706–10711. 10.1073/pnas.0502441102 16024728 PMC1180773

[B153] Miljkovitch R. Pierrehumbert B. Bretherton I. Halfon O. (2004). Associations between parental and child attachment representations. *Attach. Hum. Dev.* 6 305–325. 10.1080/14616730412331281557 15513271

[B154] Miller E. K. Cohen J. D. (2001). An integrative theory of prefrontal cortex function. *Annu. Rev. Neurosci.* 24 167–202. 10.1146/annurev.neuro.24.1.167 11283309

[B155] Morris M. W. Chiu C.-Y. Liu Z. (2015). Polycultural psychology. *Annu. Rev. Psychol.* 66 631–659. 10.1146/annurev-psych-010814-015001 25251481

[B156] Nakamura S. Sakaguchi T. (1990). Development and plasticity of the locus coeruleus: a review of recent physiological and pharmacological experimentation. *Prog. Neurobiol.* 34 505–526. 10.1016/0301-0082(90)90018-c 2202018

[B157] Nelson K. Gruendel J. (1981). Generalized event representations: basic building blocks of cognitive development. *Adv. Dev. Psychol.* 1 131–158.

[B158] Ng S. H. Lai J. C. (2009). Effects of culture priming on the social connectedness of the bicultural self: a self-reference effect approach. *J. Cross Cult. Psychol.* 40 170–186. 10.1177/0022022108328818

[B159] North A. C. Hargreaves D. J. McKendrick J. (1999). The influence of in-store music on wine selections. *J. Appl. Psychol.* 84 271–276. 10.1037//0021-9010.84.2.271

[B160] Ochsner K. N. Bunge S. A. Gross J. J. Gabrieli J. D. (2002). Rethinking feelings: an FMRI study of the cognitive regulation of emotion. *J. Cogn. Neurosci.* 14 1215–1229. 10.1162/089892902760807212 12495527

[B161] O’Connor E. E. Collins B. A. Supplee L. (2012). Behavior problems in late childhood: the roles of early maternal attachment and teacher-child relationship trajectories. *Attach. Hum. Dev.* 14 265–288. 10.1080/14616734.2012.672280 22537524

[B162] Ohman A. (2005). The role of the amygdala in human fear: automatic detection of threat. *Psychoneuroendocrinology* 30 953–958. 10.1016/j.psyneuen.2005.03.019 15963650

[B163] Ontai L. L. Thompson R. A. (2002). Patterns of attachment and maternal discourse effects on children’s emotion understanding from 3 to 5 years of age. *Soc. Dev.* 11 433–450. 10.1111/1467-9507.00209

[B164] Ortigue S. Bianchi-Demicheli F. de C. Hamilton A. Grafton S. T. (2007). The neural basis of love as a subliminal prime: an event-related functional magnetic resonance imaging study. *J. Cogn. Neurosci.* 19 1218–1230. 10.1162/jocn.2007.19.7.1218 17583996

[B165] Phua D. Y. Meaney M. J. Khor C. C. Lau I. Y. M. Hong Y.-Y. (2017). Effects of bonding with parents and home culture on intercultural adaptations and the moderating role of genes. *Behav. Brain Res.* 325 223–236. 10.1016/j.bbr.2017.02.012 28202409

[B166] Piaget J. (1952). *The Origins of Intelligence in Children* Vol. 8 New York, NY: International Universities Press, 18

[B167] Piaget J. (1976). “Piaget’s theory,” in *Piaget and his School* eds Inhelder B. Chipman H. H. (New York, NY: Springer-Verlag).

[B168] Puddy R. W. Jackson Y. (2003). The development of parenting skills in foster parent training. *Child. Youth Serv. Rev.* 25 987–1013. 10.1016/s0190-7409(03)00106-3

[B169] Purves D. Cabeza R. Huettel S. A. LaBar K. S. Platt M. L. Woldorff M. G. (2008). *Cognitive Neuroscience.* Sunderland: Sinauer As-sociates, Inc.

[B170] Pyszczynski T. Greenberg J. Solomon S. Arndt J. Schimel J. (2004). Why do people need self-esteem? A theoretical and empirical review. *Psychol. Bull.* 130 435–468. 10.1037/0033-2909.130.3.435 15122930

[B171] Quirin M. Gillath O. Pruessner J. C. Eggert L. D. (2009). Adult attachment insecurity and hippocampal cell density. *Soc. Cogn. Affect. Neurosci.* 5 39–47. 10.1093/scan/nsp042 20007241 PMC2840841

[B172] Quirk G. J. Beer J. S. (2006). Prefrontal involvement in the regulation of emotion: convergence of rat and human studies. *Curr. Opin. Neurobiol.* 16 723–727. 10.1016/j.conb.2006.07.004 17084617

[B173] Raikes H. A. Thompson R. A. (2006). Family emotional climate, attachment security and young children’s emotion knowledge in a high risk sample. *Br. J. Dev. Psychol.* 24 89–104. 10.1348/026151005x70427

[B174] Roisman G. I. Tsai J. L. Chiang K.-H. S. (2004). The emotional integration of childhood experience: physiological, facial expressive, and self-reported emotional response during the adult attachment interview. *Dev. Psychol.* 40 776–789. 10.1037/0012-1649.40.5.776 15355165

[B175] Rolls E. T. (2007). “Emotion elicited by primary reinforcers and following stimulus-reinforcement association learning,” in *The Handbook of Emotion Elicitation and Assessment* eds Coan J. A. Allen J. J. B. (New York, NY: Oxford University Press) 137–157.

[B176] Ross M. Xun W. E. Wilson A. E. (2002). Language and the bicultural self. *Pers. Soc. Psychol. Bull.* 28 1040–1050.

[B177] Sable P. (2013). The pet connection: an attachment perspective. *Clin. Soc. Work J.* 41 93–99. 10.1007/s10615-012-0405-2

[B178] Scannell L. Gifford R. (2010). Defining place attachment: a tripartite organizing framework. *J. Environ. Psychol.* 30 1–10. 10.1016/j.jenvp.2009.09.006

[B179] Scannell L. Gifford R. (2017). Place attachment enhances psychological need satisfaction. *Environ. Behav.* 49 359–389. 10.1177/0013916516637648

[B180] Schneider B. H. Atkinson L. Tardif C. (2001). Child-parent attachment and children’s peer relations: a quantitative review. *Dev. Psychol.* 37 86–100. 10.1037//0012-1649.37.1.86 11206436

[B181] Sedikides C. Wildschut T. Arndt J. Routledge C. (2008). Nostalgia: past, present, and future. *Curr. Dir. Psychol. Sci.* 17 304–307.

[B182] Sedikides C. Wildschut T. Baden D. (2004). “Nostalgia: conceptual issues and existential functions,” in *Handbook of Experimental Existential Psychology* eds Greenberg J. Koole S. L. Pyszczynski T. (New York, NY: Guilford Press) 200–214.

[B183] Sharpe T. M. Killen J. D. Bryson S. W. Shisslak C. M. Estes L. S. Gray N. (1998). Attachment style and weight concerns in preadolescent and adolescent girls. *Int. J. Eat. Disord.* 23 39–44. 9429917 10.1002/(sici)1098-108x(199801)23:1<39::aid-eat5>3.0.co;2-2

[B184] Sheppes G. Catran E. Meiran N. (2009). Reappraisal (but not distraction) is going to make you sweat: physiological evidence for self-control effort. *Int. J. Psychophysiol.* 71 91–96. 10.1016/j.ijpsycho.2008.06.006 18625273

[B185] Shweder R. A. (1999). Why cultural psychology? *Ethos* 27 62–73. 10.1525/eth.1999.27.1.62

[B186] Sierra-Mercado D. Corcoran K. A. Lebrn-Milad K. Quirk G. J. (2006). Inactivation of the ventromedial prefrontal cortex reduces expression of conditioned fear and impairs subsequent recall of extinction. *Eur. J. Neurosci.* 24 1751–1758. 10.1111/j.1460-9568.2006.05014.x 17004939

[B187] Simpson M. (1979). Daytime rest and activity in socially living rhesus monkey infants. *Anim. Behav.* 27 602–612. 10.1016/0003-3472(79)90197-0

[B188] Slough N. M. Greenberg M. T. (1990). Five-year-olds’ representations of separation from parents: responses from the perspective of self and other. *New Dir. Child Adolesc. Dev.* 48 67–84. 10.1002/cd.23219904806 2216012

[B189] Smith E. R. Murphy J. Coats S. (1999). Attachment to groups: theory and management. *J. Pers. Soc. Psychol.* 77 94–110. 10.1037//0022-3514.77.1.94 10434410

[B190] Sroufe L. A. Egeland B. Kreutzer T. (1990). The fate of early experience following developmental change: longitudinal approaches to individual adaptation in childhood. *Child Dev.* 61 1363–1373. 10.1111/j.1467-8624.1990.tb02867.x 2245730

[B191] Sroufe L. A. Waters E. (1977a). Attachment as an organizational construct. *Child Dev.* 48 1184–1199. 10.1111/j.1467-8624.1977.tb03922.x

[B192] Sroufe L. A. Waters E. (1977b). Heart rate as a convergent measure in clinical and developmental research. *Merrill Palmer Q. Behav. Dev.* 23 3–27.

[B193] Steele H. Steele M. Croft C. Fonagy P. (1999). Infant-mother attachment at one year predicts childrens understanding of mixed emotions at six years. *Soc. Dev.* 8 161–178. 10.1111/1467-9507.00089

[B194] Stern D. N. (1985). *The Interpersonal World of the Infant: A View from Psychoanalysis and Developmental Psychology.* London: Karnac Books.

[B195] Sui J. Zhu Y. Chiu C.-Y. (2007). Bicultural mind, self-construal, and self-and mother-reference effects: consequences of cultural priming on recognition memory. *J. Exp. Soc. Psychol.* 43 818–824. 10.1016/j.jesp.2006.08.005

[B196] Sullivan R. M. (2003). Developing a sense of safety. *Ann. N. Y. Acad. Sci.* 1008 122–131. 10.1196/annals.1301.013 14998878 PMC1868534

[B197] Tajfel H. (1974). Social identity and intergroup behaviour. *Information* 13 65–93. 10.1177/053901847401300204

[B198] Tajfel H. (2010). *Social Identity and Intergroup Relations.* Cambridge: Cambridge University Press.

[B199] Tam K.-P. Lau H. P. B. Jiang D. (2012). Culture and subjective well-being: a dynamic constructivist view. *J. Cross Cult. Psychol.* 43 23–31. 10.1177/0022022110388568

[B200] Taylor S. E. Klein L. C. Lewis B. P. Gruenewald T. L. Gurung R. A. Updegraff J. A. (2000). Biobehavioral responses to stress in females: tend-and-befriend, not fight-or-flight. *Psychol. Rev.* 107 411–429. 10.1037//0033-295x.107.3.411 10941275

[B201] Taylor S. E. Lerner J. S. Sage R. M. Lehman B. J. Seeman T. E. (2004). Early environment, emotions, responses to stress, and health. *J. Pers.* 72 1365–1394. 10.1111/j.1467-6494.2004.00300.x 15509286

[B202] Thompson R. A. (2000). The legacy of early attachments. *Child Dev.* 71 145–152. 10.1111/1467-8624.00128 10836568

[B203] Thompson R. A. (2006). “The development o f the person: social understanding, relationships, self, conscience,” in *Handbook of Child Psychology, Social, Emotional and Personality Development* 6th Edn Vol. 3 eds Damon W. Lemer R. M. Eisenberg N. (New York, NY: Wiley) 24–98.

[B204] Thompson R. A. (2015). “Relationships, regulation, and early development,” in *Handbook of Child Psychology and Developmental Science: Social and Emotional Development*, 7th Edn Vol. 3 ed. Lerner R. M. (New York, NY: Wiley) 201–246.

[B205] Thompson R. A. Laible D. J. Ontai L. L. (2003). Early understandings of emotion, morality, and self: developing a working model. *Adv. Child Dev. Behav.* 31 139–172. 14528661 10.1016/s0065-2407(03)31004-3

[B206] Thompson R. A. Meyer S. (2007). Socialization of emotion regulation in the family. *Handb. Emot. Regul.* 249 249–268.

[B207] Trafimow D. Triandis H. C. Goto S. G. (1991). Some tests of the distinction between the private self and the collective self. *J. Pers. Soc. Psychol.* 60 649–655. 10.1037//0022-3514.60.5.649

[B208] Triandis H. C. (2010). “Culture and psychology: a history of the study of their relationship,” in *Handbook of Cultural Psychology* eds Kitayama S. Cohen D. (New York, NY: Guilford Press).

[B209] Tulving E. (1972). Episodic and semantic memory. *Organ. Mem.* 1 381–403.

[B210] Verkuyten M. Pouliasi K. (2002). Biculturalism among older children: cultural frame switching, attributions, self-identification, and attitudes. *J. Cross Cult. Psychol.* 33 596–609. 10.1177/0022022102238271

[B211] Verschueren K. Marcoen A. (2002). Perceptions of self and relationship with parents in aggressive and nonaggressive rejected children. *J. Sch. Psychol.* 40 501–522. 10.1016/s0022-4405(02)00122-x

[B212] Verschueren K. Marcoen A. (2005). “Perceived security of attachment to mother and father: developmental differences and relations to self-worth and peer relationships at school,” in *Attachment in Middle Childhood* eds Kerns K. A. Richardson R. A. (New York, NY: Guilford Press) 71–88.

[B213] Verschueren K. Marcoen A. Schoefs V. (1996). The internal working model of the self, attachment, and competence in five-year-olds. *Child Dev.* 67 2493–2511. 10.1111/j.1467-8624.1996.tb01870.x 9022252

[B214] Vrticka P. Vuilleumier P. (2012). Neuroscience of human social interactions and adult attachment style. *Front. Hum. Neurosci.* 6:212. 10.3389/fnhum.2012.00212 22822396 PMC3398354

[B215] Wareham P. Salmon K. (2006). Mother-child reminiscing about everyday experiences: implications for psychological interventions in the preschool years. *Clin. Psychol. Rev.* 26 535–554. 10.1016/j.cpr.2006.05.001 16815615

[B216] Waters E. Cummings E. M. (2000). A secure base from which to explore close relationships. *Child Dev.* 71 164–172. 10.1111/1467-8624.00130 10836570

[B217] Waters H. S. Rodrigues L. M. Ridgeway D. (1998). Cognitive underpinnings of narrative attachment assessment. *J. Exp. Child Psychol.* 71 211–234. 10.1006/jecp.1998.2473 9878106

[B218] Waters H. S. Waters E. (2006). The attachment working models concept: among other things, we build script-like representations of secure base experiences. *Attach. Hum. Dev.* 8 185–197. 10.1080/14616730600856016 16938702

[B219] Weisner T. S. Gallimore R. Bacon M. K. Barry H. III Bell C. Novaes S. C. (1977). My brother’s keeper: child and sibling caretaking. *Curr. Anthropol.* 18 169–190. 10.1086/201883

[B220] Wong R. Y.-M. Hong Y. (2005). Dynamic influences of culture on cooperation in the prisoner’s dilemma. *Psychol. Sci.* 16 429–434. 15943667 10.1111/j.0956-7976.2005.01552.x

[B221] Wyer R. S. Jr. (2014). *The Automaticity of Everyday Life: Advances in Social Cognition* Vol. 10. London: Psychology Press.

[B222] Yap W. J. Christopoulos G. I. Hong Y. (2017). Physiological responses associated with cultural attachment. *Behav. Brain Res.* 325(Pt B) 214–222. 10.1016/j.bbr.2017.01.017 28110004

[B223] Younger J. Aron A. Parke S. Chatterjee N. Mackey S. (2010). Viewing pictures of a romantic partner reduces experimental pain: involvement of neural reward systems. *PLoS One* 5:e13309. 10.1371/journal.pone.0013309 20967200 PMC2954158

[B224] Yunger J. L. Corby B. C. Perry D. G. (2005). “Dimensions of attachment in middle childhood,” in *Attachment in Middle Childhood* eds Kerns K. A. Richardson R. A. (New York, NY: Guilford Press) 89–114.

